# SARS-CoV-2: is there neuroinvasion?

**DOI:** 10.1186/s12987-021-00267-y

**Published:** 2021-07-14

**Authors:** Conor McQuaid, Molly Brady, Rashid Deane

**Affiliations:** Department of Neuroscience, University of Rochester, URMC, 601 Elmwood Avenue, Rochester, NY 14642 USA

**Keywords:** Blood–brain barrier (BBB), Cerebrospinal fluid (CSF), Choroid plexus, COVID-19, Aging, Obesity, Hypertension, Diabetes, ACE2, MMP9

## Abstract

**Background:**

SARS-CoV-2, a coronavirus (CoV), is known to cause acute respiratory distress syndrome, and a number of non-respiratory complications, particularly in older male patients with prior health conditions, such as obesity, diabetes and hypertension. These prior health conditions are associated with vascular dysfunction, and the CoV disease 2019 (COVID-19) complications include multiorgan failure and neurological problems. While the main route of entry into the body is inhalation, this virus has been found in many tissues, including the choroid plexus and meningeal vessels, and in neurons and CSF.

**Main body:**

We reviewed SARS-CoV-2/COVID-19, ACE2 distribution and beneficial effects, the CNS vascular barriers, possible mechanisms by which the virus enters the brain, outlined prior health conditions (obesity, hypertension and diabetes), neurological COVID-19 manifestation and the aging cerebrovascualture. The overall aim is to provide the general reader with a breadth of information on this type of virus and the wide distribution of its main receptor so as to better understand the significance of neurological complications, uniqueness of the brain, and the pre-existing medical conditions that affect brain. The main issue is that there is no sound evidence for large flux of SARS-CoV-2 into brain, at present, compared to its invasion of the inhalation pathways.

**Conclusions:**

While SARS-CoV-2 is detected in brains from severely infected patients, it is unclear on how it gets there. There is no sound evidence of SARS-CoV-2 flux into brain to significantly contribute to the overall outcomes once the respiratory system is invaded by the virus. The consensus, based on the normal route of infection and presence of SARS-CoV-2 in severely infected patients, is that the olfactory mucosa is a possible route into brain. Studies are needed to demonstrate flux of SARS-CoV-2 into brain, and its replication in the parenchyma to demonstrate neuroinvasion. It is possible that the neurological manifestations of COVID-19 are a consequence of mainly cardio-respiratory distress and multiorgan failure. Understanding potential SARS-CoV-2 neuroinvasion pathways could help to better define the non-respiratory neurological manifestation of COVID-19.

## Background

In late 2019, an outbreak of a new coronavirus (CoV) was reported, which quickly became a pandemic [1]. This new CoV virus is called SARS-CoV-2 and causes the coronavirus disease, COVID-19 [2]. In the healthiest people, the majority of COVID-19 infections are asymptomatic, mild or moderate, who recovers after the infectious period, but in some cases, it can be life-threatening or debilitating. There are also concerns for those patients with some persistence symptoms after the usual infectious period (post-COVID-19 syndrome called ‘long-haulers’) [3–6]. While this virus infects people of all ages, most of those with a greater risk of requiring intense care and of dying from the infection are older individuals, and in particular men with pre-existing health conditions, such as respiratory diseases, obesity, hypertension, cardiovascular disease (CVD), and diabetes [7, 8]. Indeed, over 95% of the deaths from those infected were of patients > 60 years old and mostly men with comorbidities [7]. Also, post-infection hyperinflammatory diseases and multi-organ failure have been described as additional features of COVID-19. Thus, elderly individuals and those with co-morbidities are significant risk factors for developing severe COVID-19 symptoms [9]. Obesity, diabetes, hypertension and aging are also risk factors for systemic vascular dysfunction [10].

This novel CoV is more contagious than the previous major CoV outbreaks, which caused severe acute respiratory syndrome (SARS) by SARS-CoV (SARS-CoV-1) in 2002 and Middle East Respiratory Syndrome by MERS-CoV in 2012 [11]. The clinical manifestations of these infections are similar, since SARS-CoV-2, SARS-CoV and MERS-CoV share a high homology in their amino acids sequence [12]. Some symptoms of COVID-19 are similar to SARS, such as fever, cough, fatigue and body aches, which can include pneumonia and dyspnoea in severe cases [12]. Compared to SARS-CoV and MERS-CoV, SARS-CoV-2 have a lower case-fatality rate, but spreads more efficiently [13]. While the worldwide death rate of SARS-CoV and MERS-CoV were ~ 10% and ~ 36% of those infected, respectively, with a progressive rapid increase in testing, the current estimated death rate from SARS-CoV-2 is < 0.5% [14, 15].

While acute respiratory distress syndrome (ARDS) is mainly associated with SARS, increasing clinical evidence of non-respiratory symptoms, include anosmia (smell dysfunction), dysgeusia (taste dysfunction), multisystem inflammation, vascular inflammation and neurological symptoms, have also been associated with COVID-19. Clinical evidence shows that SARS-CoV-2 causes a Kawasaki-like syndrome in younger patients; with wide spread vascular inflammation [8], which may indicate it is also a vascular disease. Kawasaki disease, acute febrile illness, is seen in young children. The main features are fever, rash, swelling of the hands and feet, redness of the eyes, irritation and inflammation [16]. There are reports of children and young adults with COVID-19 having varied manifestations of hyperinflammatory states and/or Kawasaki-like disease. Consequently, these hyperinflammatory features involving multiply organs are described as multisystem inflammatory syndrome (MIS). While most COVID-19 cases (> 90%) in children are described as asymptomatic, mild or moderate infections, the long-term effects of this on the brain is unclear [16, 17].

The cerebrovasculature is susceptible to hypoxia; therefore cardio-respiratory failure and systemic inflammation can potentially damage the vasculature, leading to death. There is an urgent need to understand the pathogenic mechanisms of this virus so as to find solutions that will effectively diagnose, treat and prevent this disease, and the inevitable similar outbreaks, especially with the expected evolution of variants associated with these viruses. Thus, a better understanding on how SARS-CoV-2 affects the systems, the cerebrovasculature and whether there is neuroinvasion would have additional benefits in improving outcomes for infected susceptible patients in the short- and long-term, such as the so called ‘long-haulers’. The aim of the review is to provide the general readership with the breadth of information on the virus, the distribution of its main host cell receptor, the brain vascular barriers, and the pre-existing medical conditions that affect brain. The main issue is that there is no sound evidence for large flux of SARS-CoV-2 into brain, at present, to support a brain invasion compared to the lungs and respiratory pathways.

## SARS-CoV-2 and COVID-19

CoVs are a diverse family of viruses with a large host range, including humans, and are primarily associated with mild respiratory infections of the upper respiratory tract [9, 18]. Bats appear to have a large reservoir and diversity of CoVs (including HCoV-NL63, HCoV-2295, SARS-CoV, MERS-CoV and SARS-CoV-2) [18]. The current consensus is that SARS-CoV-2 likely originated from bats and was transmitted to humans via an intermediate host, possibly pangolins [19, 20]. Further work is needed to confirm this.

SARS-CoV-2, about 100 nm in diameter, is made up of a lipid membrane viral envelop that encloses the nucleocapsid, which is the genetic content of the virus that interacts with the host genetic material [21]. The lipid plasma membrane has structural proteins, namely the spike protein (SP), membrane protein, small membrane protein and hemagglutinin-esterase [21]. SP promotes attachment and facilitates entry into host cells by binding (kd ~ 5 pM) to the extracellular peptidase domain on angiotensin converting enzyme (ACE) 2 receptors (ACE2) on the plasma membrane [22]. TMPRESS 2 (transmembrane protease, serine 2) on the host cells cleaves the SP to facilitate viral entry [23]. SP may also interact with CD147 (EMMPRIN (extracellular matrix metalloprotease inducer)) in its invasion and dissemination processes [24]. The role of CD147 on SARS-CoV-2 spike protein-mediated disruption of cardiac pericyte-endothelial cells has been reported [25]. Also, CoV attachment to oligosaccharide receptors via sialic acid contributes to viral entry into host cells [26]. Glycoprotein containing sialic acid and N-acetylglucosamine was shown to mediate recombinant spike protein uptake into the murine brain [27]. However, it is unclear if CD147 or sialic acid mediates SARS-CoV-2 entry into host cells.

Thus, due to the similarity between CoVs, there are likely multiple interaction sites between SARS-CoV-2 and host cells, which may lead to the diverse symptoms of COVID-19, and also potential therapeutic targets.

### SARS-CoV-2 variants

While viruses mutate as they replicate and spread, the frequency of mutation and the characteristics of the variants vary. Mutations can be favorable, neutral or deleterious by altering the virulence and transmissibility, or produce no change in viral behavior. Since variants may affect the efficacy of vaccines, diagnostic methods, and could overwhelm the healthcare system, there is increased genomic surveillance. This has detected a number of SARS-CoV-2 variants worldwide, and there are emerging data on the characteristics of some these variants (Table 1).

**Table 1:** Known SARS-CoV-2 variants

Colloquially name	Scientific name: Pangolin Nextstrain	Defined by	Important mutations	Countries reported (first detected)	Increased transmissibility	Increased COVID-19 mortality or severity	Effect on vaccines
UK variant	**B.1.1.7** 20I/501Y.V1 VUI-202012/01	17 SNPs	-N501Y -69/70 deletion -P681H -D619G	75 (34) (United Kingdom)	Yes (~ 50–70%) [34, 35, 263–266]	Possibly [267, 268]	Oxford/AstraZeneca still effective [269]
South African variant	**B.1.351** 20H/501Y.V2	9 SNPs	-N501Y -K417N -E484K -D619G	32 (34) (South Africa)	Yes (~ 50%) [34, 35, 270]	Possibly [270]	Moderna may be less effective [271, 272]
Brazil variant	**P.1** B.1.1.28 20 J/501Y.V3	19 SNPs	-N501Y -K417T -E484K -D619G	12 (34) (Brazil)	Yes [35]	N/A	N/A
New York	**B.1.525–B.1.526** 20C	13 SNPs	-D614G -E484K -Q677H -F888L	30 (Multiple)	yes	N/A	Reduction in neutralization by monoclonal antibodies
California	**B.1.427–B.1.429** 20C/S:452R	10 SNPS	-L452R -D624G -N501Y	N/A	Yes (~ 20%)	N/A	Reduction in neutralization by monoclonal antibodies

Bold scientific name is pangolin, non-bold is Nextstrain

The UK (B.1.17), South Africa (B.1.351) and Brazil (P.1) variants are spreading in many counties, and others that will become more widespread. While these variants harbor a number of mutations, there are a few key mutation sites that may influence viral behavior. **N501Y**, an asparagine to tyrosine amino acid substitution at position 501 in the viral SP, is present in all three variants, and experimental data suggest this mutation increases its affinity for human ACE2 receptor [28]. A study in mice found that this mutation increases infectivity and sickness [28]. **D619G** is a mutation in the SP that is also shared by all 3 variants. In vitro data show that D619G increases infectivity by enhancing the SP/ACE2 receptor binding and fusion [29–31]. In animal models, D619G mutation enhanced replication rate in the upper respiratory tract and transmission [29, 32, 33]. **P681H** (found in the B.1.1.7 variant) is a mutation in the SP near the SP (S1/S2) furin cleavage site, but the biological relevance is unclear [34, 35]. **E484K** (found in P.1, B.1.351, and B.1.525–526 variants), a mutation in the receptor binding domain of the SP, reduces the ability of antibodies, collected from people who recovered from COVID-19, to neutralize the virus. This raise concerns that these mutations could pose a greater risk to the population because it may be better at reinfecting people and some vaccines might not be as efficacious [36].

The Denmark mink variant is consisted of a cluster of 5 mutations (69/70 deletion, Y453F, I692V, M1229I, S1147L), but the implications of these mutations are not well understood. However, preliminary findings indicate that this particular mink-associated variant, identified in both minks and human cases, has moderately decreased sensitivity to neutralizing antibodies [37–40].The adaptation of SARS-CoV-2 to mink, and the fear of the possibility that mink farms could be an animal reservoir for this viral variant led to the large-scale culling of minks on farms, and increased surveillance [38, 39].

A recent analysis suggests the presence of mutations in the USA from 45,494 complete SARS-CoV-2 genome sequence of COVID-19 patients [41]. Mutations P504L, Y541C, and L84S tend to fade out, while T85I, P323L, D614G, Q57H, L84S, R203K, R203K, and G204R may become more infectious. Interestingly, the *Wang *et al. paper also shows that mutant S24L shows a female-dominated pattern, and a more active immune systems than those of males in responding to SARS-CoV-2. Mutations on the spike protein, D614G and S24L are likely more infectious.

How these variants alter the effects of SARS-CoV-2 on the neurovasculature and neuroinvasion are unclear. Some of these mutations alter the SP, and increased the affinity for the ACE2 receptor. However, it has been demonstrated, albeit with a different receptor, that high affinity actually decreased the amount of transcytosis across the endothelium, as the cargo ended up in the lysosomal pathway [42]. If there is a non-receptor mediated entry pathway into brain that is not specific then the variants should not affect entry, but may alter its effects on brain.

## Evolution of COVID-19 symptoms: outlined

**Fig. 1:**
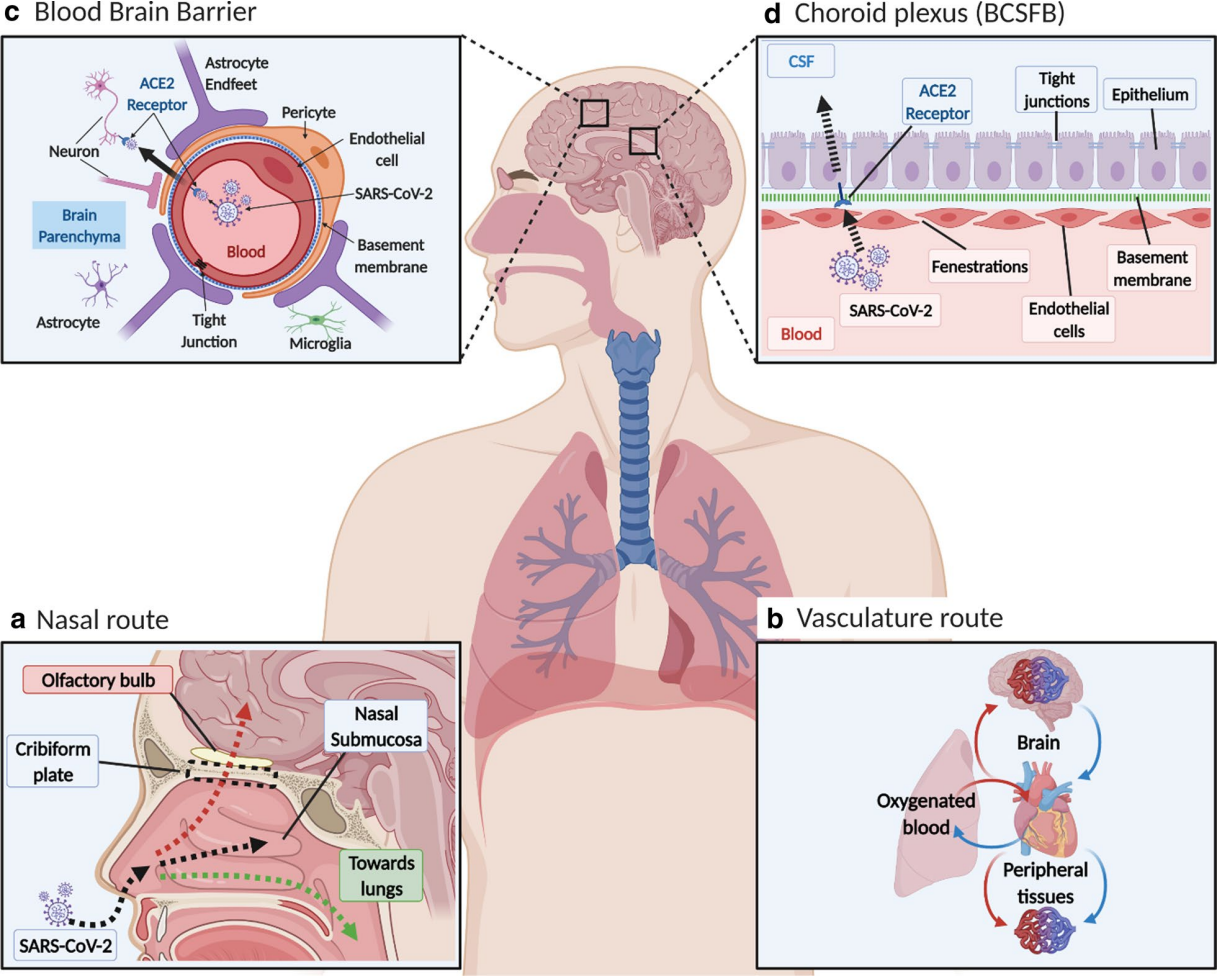
SARS-CoV-2 distribution in blood and possible entry into brain across the CNS vascular barriers. **a** Nasal entry. SARS-CoV-2 enters the nasal cavity as droplets, and (1) enters the airways with the inspired air, (2) traffic into the nasal sub mucosa via the highly vasculature of the nose and enters the blood and/or lymphatics and (3) may get access to the olfactory nerves and thus olfactory bulb by going upstream, but to date there is no sound data to viral entry into brain. **b** Vasculature entry. After it enters the lungs it may cross the thin alveolar membrane and enters the blood to access all organs, including brain, but there is no evidence that ACE2 mediate viral entery into brain. **c** Blood brain barrier (BBB). This is a highly specialized structure at the interface between the blood and the brain. It is formed by tight junctions at the endothelial cells and forms part of a complex cellular structure known as the neurovascular unit (NVU). The NVU is the functional unit of the BBB and is composed of multiple cells including, pericytes, astrocytes, microglia and neurons interacting with the endothelial cell, as well as the basement membrane, which all can affect the barrier properties. ACE2 is expressed both on endothelial cell and pericytes as well as some neurons in the brain, but there is no evidence that ACE2 mediate viral entery into brain. **d** Choroid plexus. This is at the interface between the blood and the CSF, known as the blood CSF barrier (BCSFB). The endothelial cells of the choroid plexus are leakier than in the BBB, with gaps known as fenestrations. This allows for easier movement from the blood, but the epithelium cells at the apical side are more tightly knitted together (tight junction at the apex of the epithelium), and prevent entry into CSF [261, 262], as effective as the cerebral capillaries. The choroid plexus epithelium expresses ACE2 and this acts as a possible way for SARS-CoV-2 entry into CSF and then brain parenchyma, but there is no sound evidence for this

The evolving events that lead to the development of COVID-19 symptoms are shown in Fig. 1. SARS-CoV-2 enters the body mainly as droplets during inhalation, and infiltrates the nasal and buccal cavities to gain access to the nasal mucosa and the respiratory tract, which may explain the dysgeusia and anosmia effects [43]. The virus infects the epithelium that lines the surfaces of the trachea, bronchi, bronchioles and alveoli.

The innate immune system through pathogen recognition receptors (PRRs), such as the Toll-like receptors (TLRs) and the RNA helicases, detect pathogen-associated molecular patterns (PAMPs) on viruses and trigger an antiviral response. TLRs are expressed in dendritic cells (DCs) and macrophages but can be found in nearly all human cells.

E.g., brain endothelial cells express TLR4 that can elicit proinflammatory response [44]. The TLR2/4 is expressed in the choroid plexus and circumventricular organs [45]. Equine encephalitis virus interacts with TLR4 and disrupt BBB permeability [46]. It is tempting to think that SARS-CoV-2 can also interact with these receptors. Depending on the viral load, this system is effective in eliminating the virus with minimal local and systemic inflammation (for details see, e.g., [47–54]). Thus, an effective and robust immune response can alter the outcomes of the COVID-19 infection, and contribute to a better one, such as those seen for the asymptomatic, mild and moderate cases [55].

The innate immune system plays a pivotal role by secreting mucus along the respiratory tract, which stimulates coughing and the action of cilia to remove it. The alveolar epithelium lacks mucocilliary properties but has resident granulocytes and macrophage that interacts with the virus to raise an alarm; the inflammatory response, which mobilizes and direct the body’s defense mechanisms to the sites of viral invasion (the lungs). Cytokines (e.g., IL-1, IL-6 and TNF-α) are released to stimulate the immune response, mobilize the defense mechanisms, and increase body temperature by altering the set-point in the hypothalamus [56–58]. Neutrophiles are the first immune cells to be directed to the site of infection and play a crucial role in defense process, such as phagocytosis and neutrophil extracellular traps [47, 48, 51, 59–62]. Additionally, macrophages can phagocytose SARS-CoV-2 [63, 64]. Anti-viral cytokine (interferon (IFN)) is also secreted [63, 64]. T-cells and B-cells are also stimulated, and later B-cells produce antibodies specific to the viral antigens, which can be used as evidence of SARS-CoV-2 infection, and as a possible therapy.

In response to the infection, other cells, such as mast cells, in the lungs are stimulated to release histamine, which contributes to the increased blood flow and capillary permeability leading to fluid accumulation [65]. As this increase, the alveolar can no longer perform the essential function of blood oxygenation and removal of carbon dioxide. Consequently, this decreases arterial blood pO_2_ and increased pCO_2_, which stimulates chemoreceptors in the carotid bodies, at the bifurcation of the common carotid artery, and in the central nervous system (CNS). This attempts to increase blood oxygenation by increasing ventilation and cardiac output, but due to cardio-respiratory failure, patients require a respirator to maintain this function.

## ACE2: a key player for SARS-CoV-2

**Fig. 2:**
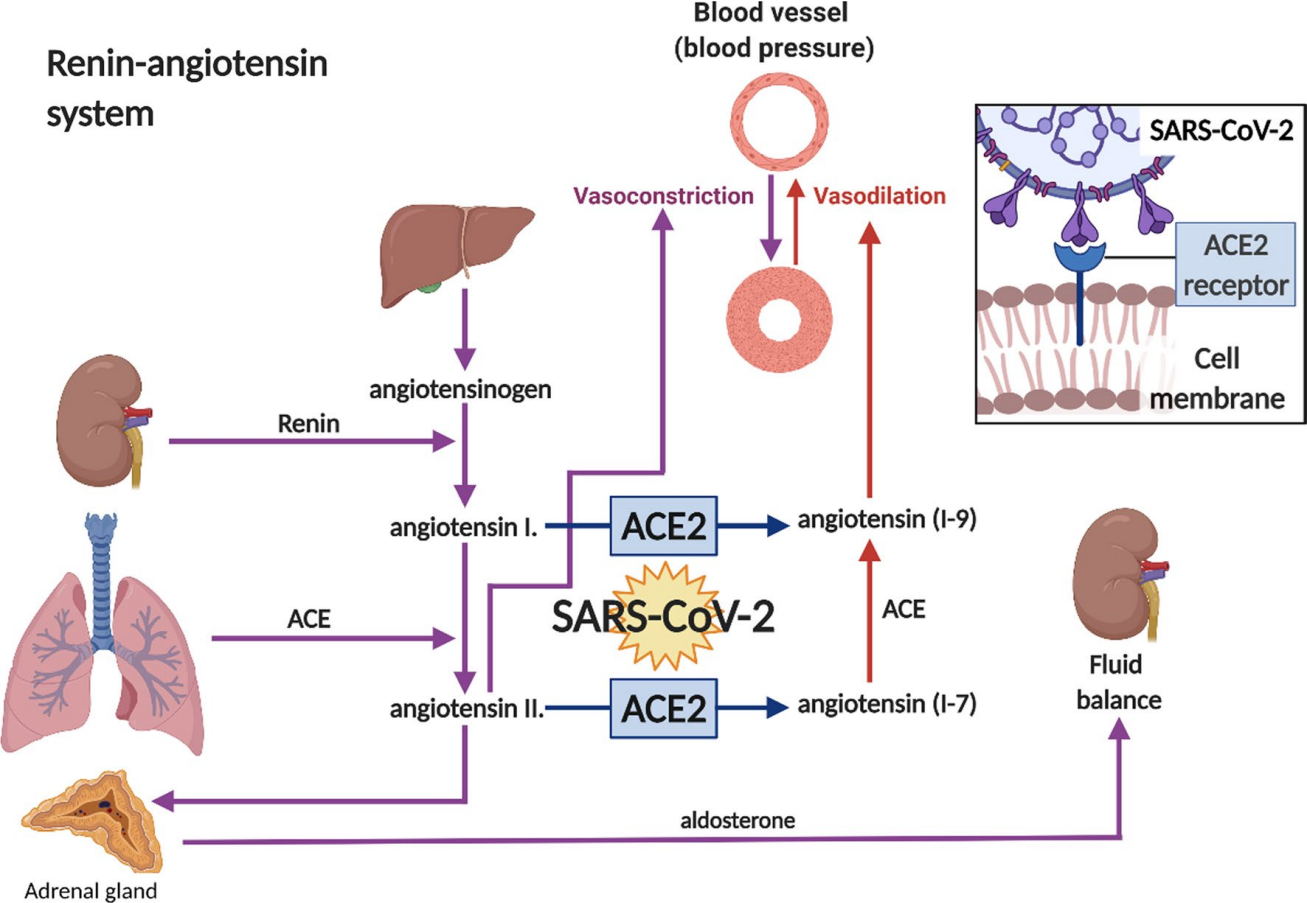
Renin-angiotensin system. ACE2 is exploited by SARS-CoV-2 when infecting host cells. Renin, secreted from the kidneys in response to changes in blood flow and pressure, catalyses the conversion of the plasma protein called angiotensinogen into angiotensin I, angiotensin converting enzyme (ACE) converts angiotensin I into angiotensin II. Angiotensin II acts via receptors in the adrenal gland, causing the secretion of aldosterone, which causes the kidney to reabsorb salt and water. Angiotensin II is a powerful vasoconstrictor. ACE2 activity alters the ratio between angiotensin I to angiotensin (1–9) and angiotensin II to angiotensin (1–7). Angiotensin (1–7) can be converted to angiotensin (1–9) by ACE, and both have been shown to cause vasodilation. It has been shown that SARS-CoV-2 spike protein binds to the ACE2 receptor and shift the balance towards angiotensin II

ACE2, a multifunctional nonocarboxypeptidase with one zinc-binding motif, plays a protective role by counteracting the effects of ACE (Fig. 2). It also hydrolyzes other peptides, such as apelin, an inotropic and cardioprotective agent [66], and kinins, which are vasoactive peptides [67]. In the intestine and kidney, ACE2 is involved in regulating amino acid transport[68]. ACE2 levels are reduced with aging, possibly due to increased activity of a disintegrin and metalloprotease17 (ADAM17) [69].

There are two forms of ACE2, membrane bound (mACE2) and the soluble circulating active extracellular domain (sACE2) [70]. During normal physiological function, and SARS-CoV-2 infection, CD147 and ADAM17 cleaves the extracellular domain of the mACE2 and release sACE2 into blood [71–76]. sACE2 levels are similar in children of both genders < 12 years old, but in older adults this is increased greater in males than females [77]. Interaction of sACE2 with SARS-CoV-2 could act like a decoy receptor, which may attenuate SARS-CoV-2 interaction with mACE2. sACE2 may also distribute SARS-CoV-2 to all organs, including liver and kidneys, for, possibly, elimination. Soluble ACE2/SARS-CoV-2 binding domain or its analogous could be used to sequester blood SARS-CoV-2 and to minimize its binding to mACE2. Since SARS-CoV-2 causes the loss of mACE2 it may increase angiotensin II effects. Thus, sACE2 levels could modulate COVID-19 severity, and also contributes to the benefits of convalescence plasma therapy.

### ACE2 main beneficial functions

ACE2’s main function is to regulate arterial blood pressure (BP) by changing total peripheral resistance to blood flow and fluid balance [14, 23, 78]. This is part of the well-known renin-angiotensin system (RAS) (Fig. 2). While the components of RAS pathways are present in the brain and the periphery, they do not directly interact, but cooperate via centers in the brain to maintain blood pressure and plasma volume [14]. RAS components are widely expressed in the brain, especially in regions that regulate the cardiovascular system (CVS) and osmoregulation, including the hypothalamic paraventricular nuclei, supraoptic nuclei, ventrolateral medulla and the solitary nucleus [79]. Angiotensin-11, generated via ACE, increases BP by increasing vasoconstriction of arterioles and fluid retention by increasing the secretion of aldosterone, in addition to inflammation, oxidative stress and injury. Degradation of angiotensin-1 and angiotensin-11 by ACE2 generates angiotensin (1–7) and angiotensin (1–9), which reduce BP, by increasing vasodilation and reducing plasma volume, inflammation, oxidative stress and injury [80]. For example, the phenotype of ACE2^−/−^ mice includes hypertension, behavioural dysfunction, impaired serotonin synthesis and neurogenesis [81]. In addition, ACE2 polymorphism is associated with essential hypertension [82]. Increased levels of brain ACE2 are associated with delay in cognitive decline [83]. Thus, ACE and ACE2 have a synergistic effect (Fig. 2). Inactivation of ACE2 as seen with SARS-CoV-2 infection could exacerbate angiotensin-11 adverse effects, such as inflammation, and perhaps, contribute to COVID-19 adverse effects. Reduced ACE2 levels are associated with sperm-related infertility [84], pulmonary hypertension [85], kidney disease [86] and acute lung injury [87, 88]. In contrast, estrogen is reported to increase ACE2 activity [89], and protects against CVD. Thus, ACE2 has a wide range of beneficial effects, but is not directly associated with initiation of inflammation.

### ACE2 expression

ACE2 is ubiquitously expressed on the cell membrane in many tissues, including those of the respiratory and cardiovascular systems, gastrointestinal tract, testes, kidneys, choroid plexus, placenta and bladder [14, 23, 78]. It is expressed in the heart (myocytes), vascular endothelial cells, and vascular smooth muscles cells of arteries and venules [21]. ACE2 is highly expressed on type 1 and type 11 epithelial cells and bronchiolar epithelium [90, 91]. In addition, it is also expressed on the oral mucosa [92].

ACE2 protein and mRNA are expressed in rodent brains [93, 94]. ACE2 mRNA is present in the cortex, striatum, hippocampus and brain stem [95]. It is expressed in cultured glial cells, although it is unclear if this occurs in non-cultured glial cells [96]. ACE2 is mainly expressed in the cytoplasm of neurons and in brain regions associated with regulation of the CVS, blood pressure (BP) and the autonomic nervous system [97]. More recently, it was reported that the brain endothelium expresses ACE2 as the protein [98] and as the RNA-seq [99]. ACE2 as well as other facilitators are present in arterial and venous endothelial cells and arterial smooth muscle cells [90]. The brain vascular endothelium shows a high expression of the SARS-CoV-2 protease cathepsin B (CTSB) but not for TMPRSS2. CTSB is expressed in veins and capillaries in brain vasculature [100]. ACE2 is highly expressed on pericytes [101]. The expression of ACE2 in cerebral endothelial cells is still obscure. It was suggested that ACE2 is present on human cerebral micro vessels using immunohistochemistry [98] and brain endothelial cells [90]. Confirmation of the vessel type and whether ACE2 is on cell membrane or intracellular are needed. In general, ACE2 has a protective role for many organs, but the significance of ACE2 role on the in vivo cerebral endothelium compared to that of peripheral organs in COVID-19 needs to be determined.

### ACE2/SARS-CoV-2

ACE2 is the primary mechanism of SARS-CoV and SARS-CoV-2 entry into host cells [78]. Although SARS-CoV-2 is similar to beta Coves, the receptor binding site is more comparable to SARS-CoV, but binds to the host cell receptor with a higher affinity than that of SARS-CoV [102]. The wide expression of mACE2 receptor suggests that many systems will be affected by SARS-CoV-2. Thus, critical cases of COVID-19 infection commonly manifest as cardiopulmonary symptoms and in severe cases, advanced into multiorgan failure and sepsis, hypothesized to be as a result of a “cytokine storm”. However, some cells without a detectable expression level of ACE2, such as hepatocytes have been shown to be infected by SARS [103]. The infectious agents of SARS and MERS were also reported in the CNS where the expression level of ACE2 or DDP4 (MERS binding receptor) is very low under normal conditions [103, 104]. Thus, there are other mechanisms that may facilitate viral entry in these organs.

## CNS vascular barriers: restrict trafficking into and out of brain

The CNS is unique in that it is enclosed within its vascular barriers, and has cerebrospinal fluid (CSF) continuously circulating within it. The brain has a high demand for oxygen and metabolites, and normally receives about 20% of the cardiac output [105], and as a result is more sensitive to hypoxic and ischemic conditions. The total length of the human brain microvasculature is approximately 600 km with a surface area of about 15–25 m^2^ [106, 107]. It is estimated that each neuronal cell body is within 10–20 µm from the nearest capillary, which allows for rapid diffusion of metabolites and nutrients [108]. The cerebrovasculature is an important metabolic regulator for the CNS [109].

### Vascular barriers

**Fig. 3:**
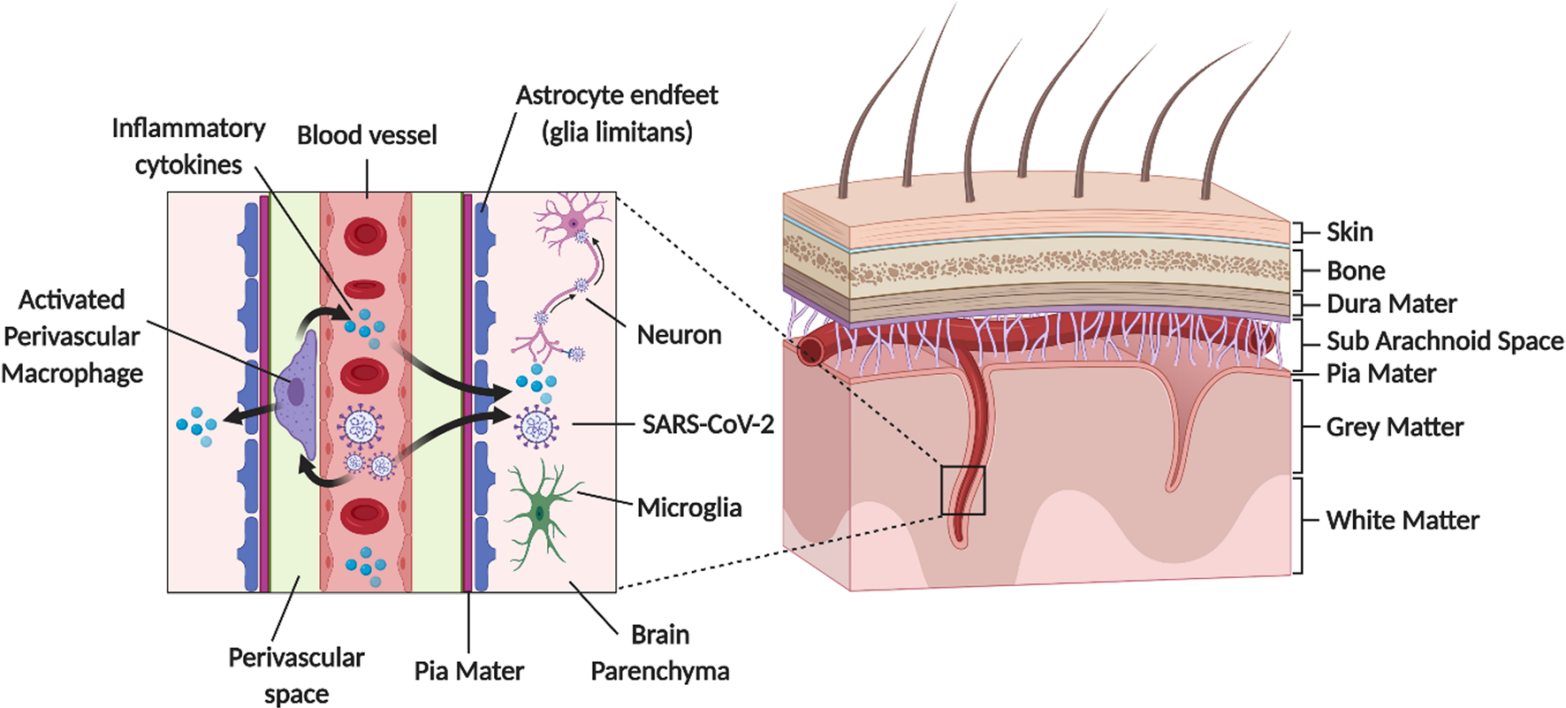
Schematic diagram showing perivascular SARS-CoV-2 entry into brain. penetrating arterial vessel and a magnified region (boxed area) showing possible access site of the virus via the perivascular space. This could activate perivascular macrophage leading to neuroinflammation

**Fig. 4:**
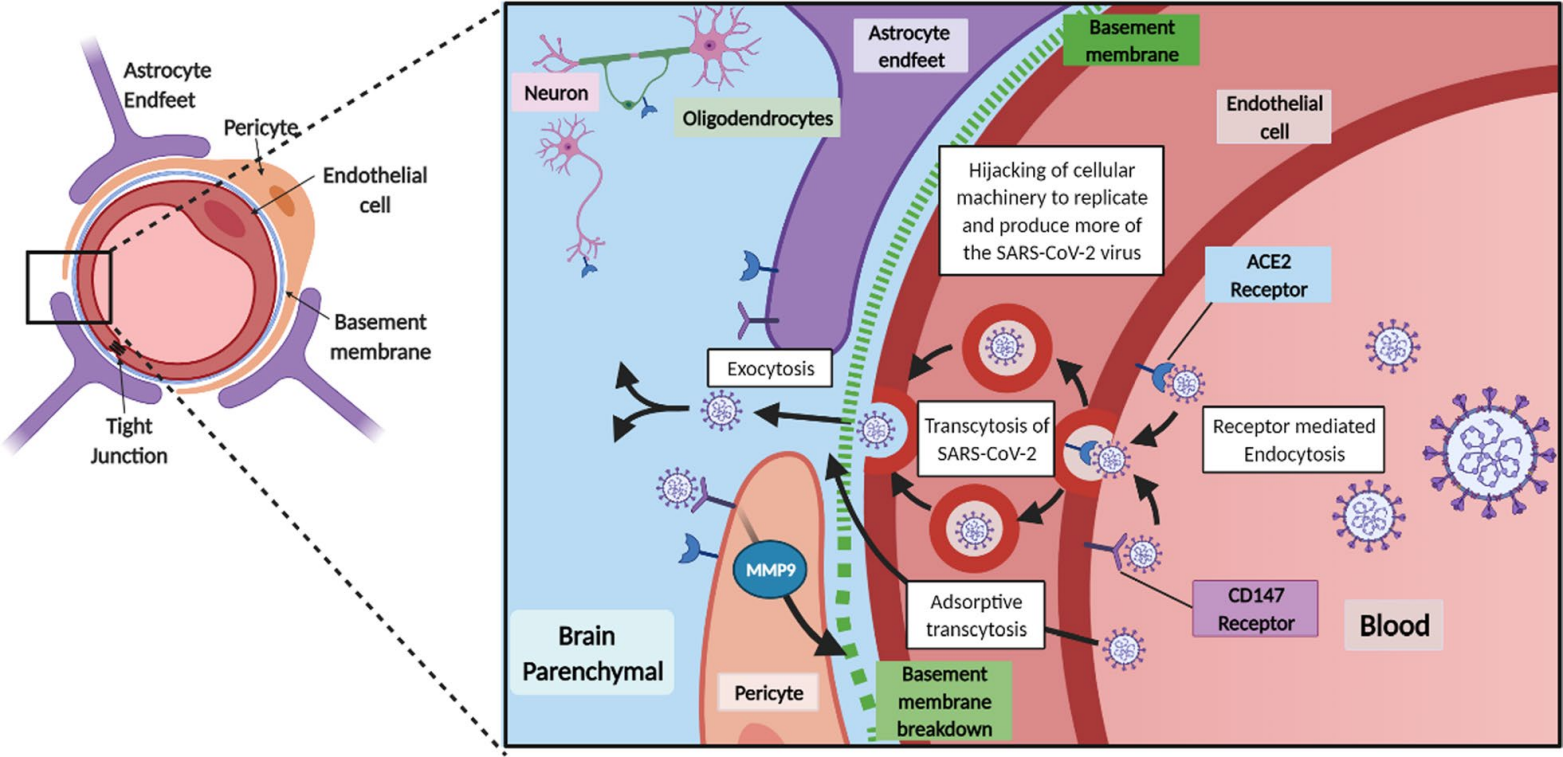
SARS-CoV-2 interaction and transport across the BBB. Normal and infected neurovascular unit (NVU). A normal BBB restricts and controls the entry of substances in and out of the brain. Tight junctions between endothelial cells restrict the movement via the paracellular route for small molecules, like ions (e.g., potassium, sodium). Along with this tight barrier there is also low levels of pinocytosis, numerous transporters for select molecules, and efflux pumps to remove substances from the brain into the blood. In an infected/diseased BBB, there is the possibilities that tight junctions loosen, allowing larger molecules to pass via the paracellular route into the brain, decrease in efflux pumps and transporters as well as increased pinocytosis altering the balance across the barrier. Basement membrane breakdown on the abluminal surface can cause increase the barrier permeability. Viral infection can cause the release of cytokines, which can alter the integrity of the BBB and increase immune cell penetration into the brain

**Fig. 5:**
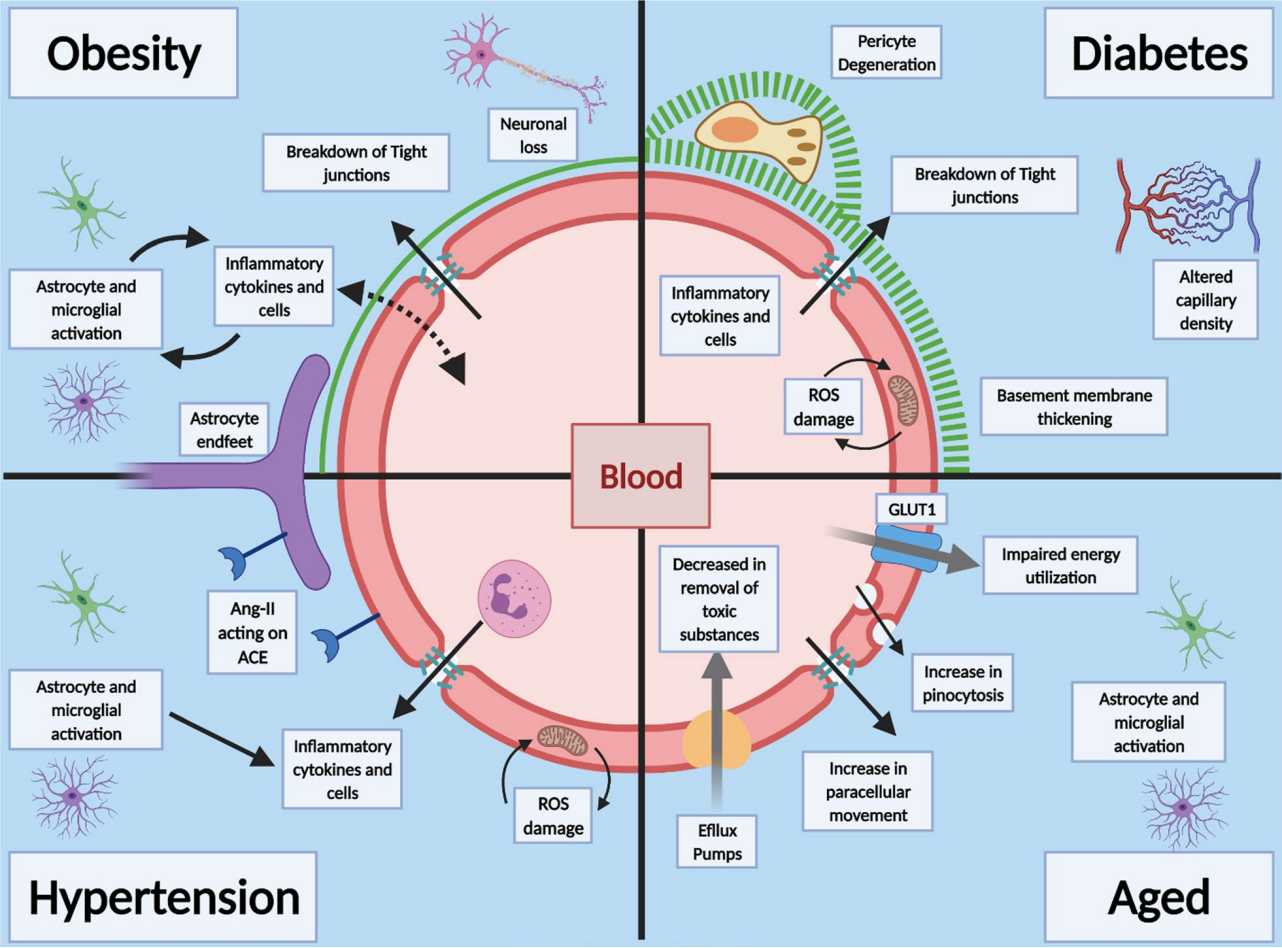
Prior health conditions effects on the BBB. Obesity is linked to increase in inflammatory cytokines which can lead to activation of astrocytes and microglia and death of neuronal cells, leading to wide spread neuro-inflammation, tight junction breakdown and damage to the CNS. Diabetes and hyperglycaemia cause pathological changes to the BBB, including increased capillary density, thickening of the basement membrane, breakdown of tight junctions and increase in paracellular diffusion. Pericytes are also shown to degenerate and aid in the decrease in barrier properties. Hypertension is linked to the RAS system and Ang-11 levels, increase in inflammatory cytokines and Reactive oxygen species (ROS) damage to endothelial cells and activation of microglia and astrocytes. Age has widespread effects on the BBB, decrease in barrier functions, decrease in efflux pumps and impaired energy utilization. There is also increased movement across the BBB by increase in paracellular movement, increase in pinocytosis leading to astrocytes and microglia activation

The vascular barriers at the blood–brain interface (the blood brain barrier, BBB) and blood-CSF interface (blood CSF barrier; the choroid plexuses) restrict the free diffusion of polar molecules into and out of the brain, while essential metabolites are transported into brain and/or into CSF [105, 110–114]. The physical site of the BBB and blood CSF barrier is the tight junctions between endothelial cells and epithelial cells, respectively [115, 116], which restricts paracellular diffusion. The endothelium of the cerebrovasculature is at the interface between the blood and brain, but there are several cell types on the abluminal side of the endothelium. These include pericytes, astrocyte end-feet and microglia, which maintain and regulate the BBB, and couples regional cerebral blood flow to meet metabolic demands [105]. Collectively these cell types make up the neurovascular unit (NVU) (Figs. 3, 4, 5) The vascular barriers also contribute to reducing the traffic of leukocytes, and microorganisms, such as viruses, from entering the brain as these require specific receptors for attachment [117].

The ependymal layer, which is an epithelium that lines the floor of the ventricles at the interface of CSF and brain parenchyma, provide regional structural and functional specializations [118, 119]. Unlike the modified ependymal cells that form the epithelial cells of the choroid plexus (tela choroidea), the epithelium between CSF and brain parenchyma constitutes a partial barrier, since most of these cells lack the tight junctional proteins. These ependymal epithelial cells are mainly joined with adherens and gap junctional proteins [120–124]. Thus, the barrier between the epithelial cells that separates brain parenchyma and CSF is not as effective as that at the blood-CSF interface [125, 126], and small water-soluble dyes, administered into CSF, is readily detected in brain [120, 122].

### CSF

CSF is mainly produced within the cerebral ventricles by the choroid plexuses, and circulate from the lateral ventricles to the subarachnoid space, around the brain, spinal cord and within the spinal canal, before ultimately draining at multiple outflow sites into blood [127–129]. This is the third circulation or pseudo lymphatics of the brain since the brain parenchyma lacks lymphatic vessels [130–134]. A major CSF drainage pathway is via the olfactory bulb, across the cribriform plate and towards the cervical lymphatic nodes, and the spinal cord [135–138]. There is no significant flow of CSF from the subarachnoid space to the brain parenchyma, under near normal experimental conditions (e.g., [138]). The flowing CSF maintains intracranial pressure and contributes to the removal of metabolites [127, 139].

Some viruses, such as Zika, human immunodeficiency virus (HIV) and human polyomavirus, enter CSF via the choroid plexus [140–143]. SARS-CoV-2 interact with organoid human pluripotent stem cell of the choroid plexus [144]. However, it’s unclear on whether SARS-CoV-2 crosses the choroid plexus in humans to significantly contribute to neuroinvasion and inflammation. The virus RNA was detected in the CSF of a COVID-19 patient with meningitis [145]. In contrast, the SARS-CoV-2 RNA was not detected in the nasopharyngeal swab but was detected in the CSF [146]. In addition, there are reports of the presence of SARS-CoV-2 in the CSF[147] and other reports showing that it is not in CSF [12, 148]. While there are indications that the virus is present in the olfactory mucosa, using autopsy samples, it’s still unclear if SARS-CoV-2 invades the human brain to contribute significantly to the disease outcomes.

Leukocytes traffic across the NVU and choroid plexus to enter brain parenchyma. Although a robust immune response can be mounted in the CNS, this organ is normally immunologically quiescent [149]. The majority immune cells found in the CSF of healthy individuals are T cells that have entered the CNS through the BBB, choroid plexus and meninges. Intravenously administrated fluorescently labelled lymphocytes are detected in some of these regions, in mice [50, 54, 150–152]. Some viruses, such as Zika and influenza A, can enter brain associated with monocytes, which can be a repository of these viruses [153–155];. While SARS-CoV-2 activates systemic monocytes [156], there is no evidence that it is involved in SARS-CoV-2 traffic into brain.

## COVID-19 and neurological symptoms

COVID-19 clinical signs are mainly associated with respiratory symptoms, but there is evidence that there are adverse neurological symptoms, including anosmia, dysgeusia, headaches, nausea and vomiting [21, 96]. These symptoms may have short- and long-term adverse effects on infected people, especially those with pre-existing health conditions. Earlier observations of COVID-19 infection found about 36% of patients had neurological manifestations [157]. This was more common in patients with severe cases and particularly for those with preconditions, such as CVD, including ischemic and haemorrhagic strokes and impaired consciousness [79]. Older patients with CVD and risk factors, such as hypertension, diabetes and higher levels of C-reactive protein compared to patients without CVD were at greater risk of severe COVID-19 symptoms and poorer prognosis [158]. Others reported that COVID-19 patients had clinical presentations of large-vessel ischemic stroke in young patients, acute necrotizing encephalopathy, encephalitis, meningitis, headaches and dizziness [81–91]. Patients with Parkinson's disease (PD) appear also to be affected by COVID-19 by exacerbating motor symptoms, such as tremor and dyskinesias, and reducing the efficacy of dopaminergic medication [159–162]. There is emerging evidence that there are psychological and behavioural changes (mental health) associated with COVID-19 infection due, perhaps, to the mitigating efforts to minimize the viral transmission, such as the isolation. There are changes in anxiety, sleep, mood, alcohol consumption and drug abuse [163, 164], and stress from the treatment of severe cases, post-traumatic stress syndrome and depression[165]. However, it’s unclear if these clinical neurological presentations seen in COVID-19 patients are due to the virus entering brain or as a consequence of cardio-respiratory and multiorgan failure.

In some patents, COVID-19 is associated with thrombotic vascular events, such as strokes [166, 167]. The incidence of strokes is 7.6-fold greater with COVID-19 compared to that of influenza infections [168]. There is a similar sevenfold increase in the incidence of large vessel stroke in young people with COVID-19 compared to the previous year’s cases [169–173]. The incidence of cerebrovascular disease in COVID-19 patients is estimated at 1–6%, [169, 174], and possible mechanisms include cytokine storm, hypercoagulation, endotheliitis and endotheliopathy. Viral particles are associated with the cerebrovasculature and the endothelium of other organs [169, 174, 175]. In severe endothelium injury; there is vascular thrombosis, microangiopathy and angiogenesis [176]. The raised plasma levels of von Willebrand factor and soluble thrombomodulin may indicate vascular injury [177]. Infiltration of leukocytes and intravascular coagulation may be due to compromised vascular integrity [175].

Neurological disorders were also associated with SARS and MERS. SARS was associated with seizures, myopathy, rhabdomyolysis and viral RNA in CSF and brain tissue [178, 179]. MERS was also associated with neuropathy, delirium, acute cerebrovascular disease, confusion and seizure [180]. With the similarities between animal and human CoVs, both molecularly and symptomatically, possible mechanism for how SARS-CoV-2 behaves can be modeled. CoVs have been shown to invade, infect, and induce neurological-like disease in animal models of SARS and MERS [181–184]. It appears that neurotropism is a common feature of CoVs, and such neuroinvasion propensity of CoVs have been documented in almost all of the beta-CoVs (SARS, MERS, HCOV-229E, OC43) [103].

The impact of the SARS-CoV-2 and COVID-19 on CNS and the cerebrovasculature may be a minor and/or a secondary issue of the systemic inflammatory disease caused by the virus. The mortality of the disease is mostly due to the failure of lung, cardiovascular and kidney functions of the patients with underling diseases or those with immune dysfunction. A few cases may die from stroke as a result of thrombosis and thrombus embolization, but this could be a significant number of deaths from COVID-19-related neurological consequences, since the number of deaths from this infection is high. Currently there aren’t data that SARS-CoV-2 impacts the cerebrovasculature of mild or asymptomatic cases as there isn’t many of these cases that have been hospitalized. Further studies are needed.

## How could SARS-CoV-2 enter the brain?

The exact mechanism of SARS-CoV-2 neuroinvasion is unclear. The main mode of CoV entry into the body is inhalation, and thus, the viruses access the nasal and buccal cavities. Viruses could enter the brain by retrograde transport via sensory nerve endings within these regions, such as the cranial olfactory and trigeminal, and the autonomic nervous system [98, 185]. In addition, virus that enters blood from the infected lungs may interact with the cerebrovasculature [27] and/or at the blood-CSF barrier, the choroid plexus [186]. Virus associated with leukocytes, such as monocytes, may enter the brain via receptors, such as advanced glycation endproducts (RAGE) and platelet endothelial cell adhesion molecule-1 (PECAM-1; CD31) [64, 187]. There is also the possibility of viral neuroinvasion via the gastrointestinal tract [188].

### Olfactory bulb

SARS-CoV-2 may access the olfactory epithelium and penetrate the cribriform plate to enter the olfactory bulb, and from there spreads within the CNS. It can infect neurons or non-neural cells via ACE2 and/or TMPRSS2 receptors and transported along the olfactory nerve [189]. SARS-CoV-2 interaction with the olfactory mucosa may explain the anosmia or hyposmia seen in COVID-19 patients. In addition to the olfactory nerve, it is possible the virus can use other nerves, such as the trigeminal and the vagus, which innervate the buccal cavity, respiratory tract and lungs [190, 191]. SARS-CoV-1, another CoV, also shows a transneural penetration through the olfactory bulb in a mouse model and thus SARS-CoV-2 might behave similarly [183]. (Figs. 3, 4).

### Cerebrovasculature

Since the CNS vascular barriers are likely compromised in the severely infected patients and in the aging brain, especially in the more susceptible patient groups, SARS-CoV-2 interaction at the cerebrovasculature may potentiate its dysfunction. The leaky cerebrovascular can cause cerebral edema [192]. The local increase in interstitial pressure would decrease blood flow to the region, leading to neuronal dysfunction and cell death [105, 192]. SARS-CoV-2 induced inflammation of the meningeal cells could increase influx of fluid, which may activate resident perivascular macrophages and parenchymal microglia leading to aggravation of cerebral inflammation (Fig. 3). SARS-CoV-2 spike proteins interact with the in vitro models of the BBB[98], and slowly cross the murine cerebrovascularture via adsorptive transcytosis that is independently of ACE2 (27; 281). Thus, further studies are needed to support significant neuroinvascion as the explanation for the neurological symptoms associated with COVID-19.

There are reports of possible SARS-CoV-2 neurotropism but further studies are needed to establish whether this is related to the degree of infection (viral load), the time this occurs in COVID-19 progression, conditions that may contribute to this and the frequency of its occurrence. Also, it is unknown whether this is due to the virus crossing the normal CNS vascular barrier to cause neuroinvasion or via the dysfunctional barriers as a consequence of pneumonia induced-hypoxia. There are reports of encephalitis, which was not due to COVID-19-induced hypoxia [193] and brain cortical hyperintensity, as seen in MRI images, which may be due to viral infection [146, 194]. The virus was detected in the cortical brain tissue, which may suggest it enters brain [195]. SARS-CoV-2 is detected in the olfactory mucosa [196].

## How SARS-CoV-2 could spread within the brain

**Fig. 6:**
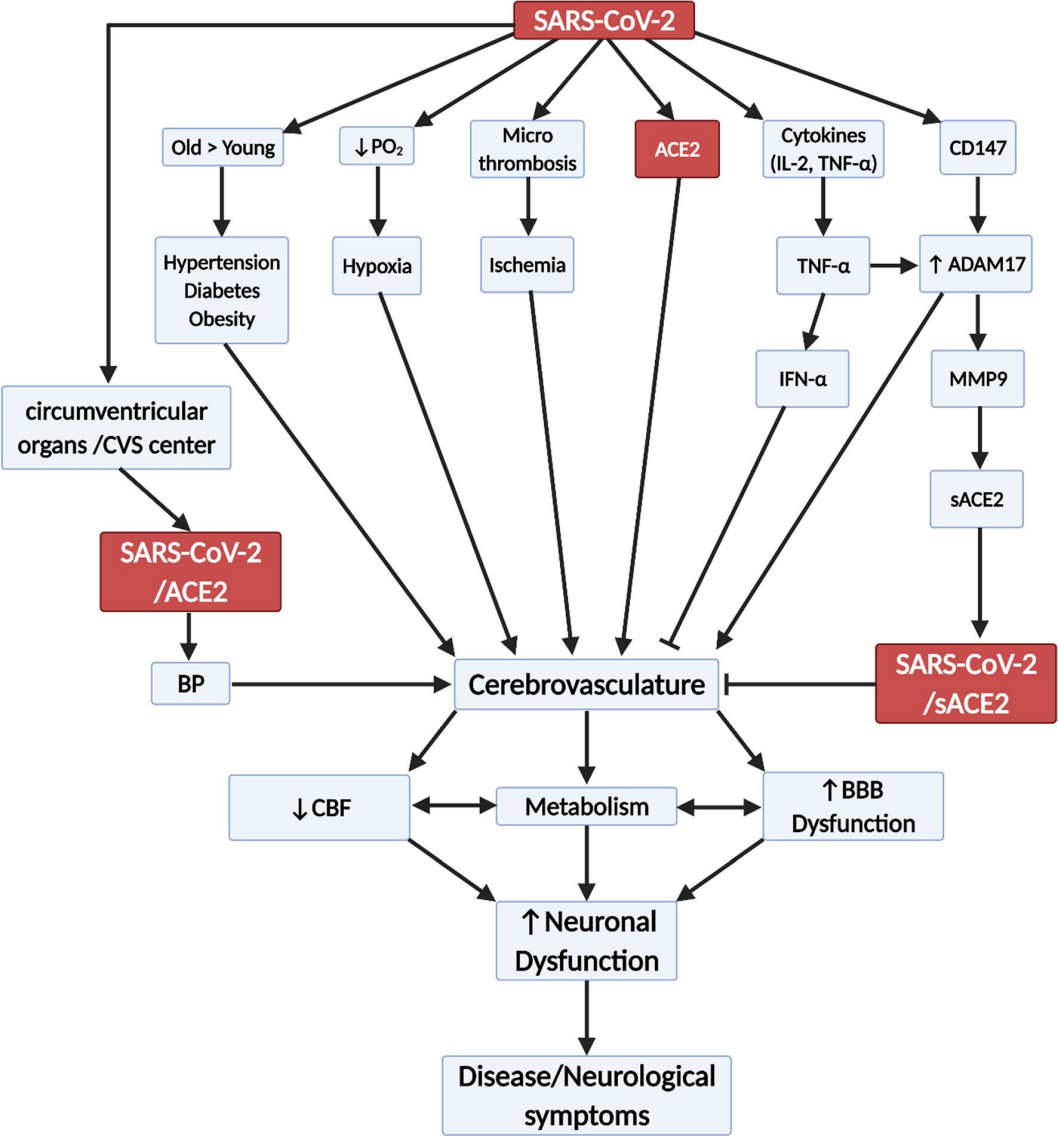
Flow diagram showing possible effects of SARS-CoV-2 on the cerebrovasculature. CBF (cerebral blood flow), BBB (blood brain barrier), ACE2 (angiotensin converting enzyme 2), sACE2 (soluble ACE), IFN (interferon), CVS (cardiovascular centre), IL-1 (interleukin 1), TNFα (tumour necrosis factor α), CD147 (EMMPRIN (extracellular matrix metalloprotease inducer), ADAM17 (disintegrin and metalloprotease17), MMP9 (matrix metalloprotease 9), BP (blood pressure) and pO_2_ (partial pressure of oxygen)

Within the brain, SARS-CoV-2 may interact with and spread through the ACE2, other facilitator receptors or by adsorptive uptake by cells. In SARS patients, SARS particles were located almost exclusively in the neurons [103]. The brainstem was heavily infected by SARS and MERS [103], and thus, all CoVs may invade the brain [103]. Neurons can then take up the virus, which then binds to intracellular ACE2 [97]. ACE2 receptor is expressed in both neurons and glia cells [195], but mainly in the cytoplasm of neurons [97]. Most studies used in vitro models to determine whether SARS-CoV-2 infect neurons or inferred that there is a potential of infection by assessing the presence of ACE2 [197, 198]. However, SARS-CoV-2 was detected in cortical neurons of infectious patients [199]. This may explain the presence of SARS in neuronal cells. Trans-synaptic transfer has been documented for other CoVs, such as HEV67 [103] and avian bronchitis virus [96]. Murine CoV can replicate and cause direct lysis of oligodendrocytes and demyelination in the CNS during the acute phase[200, 201]. In mice, infected intranasally with a large load of neuro-virulent strains of HCoV-OC43, it entered the CNS via the olfactory nerves with subsequent trans-neuronal retrograde dissemination to distant connections of the olfactory bulb, and the pyriform cortex and brainstem [201, 202]. In MERS infected mice, the presence of the virus in the CNS was associated with high mortality [103]. Neuroanatomic interconnections indicate that the death of infected animals or patients may be due to the dysfunction of the cardiorespiratory center in the brainstem [103]. It was suggested that lipids may play a role in SARS-CoV-2 in brain [203]. However, there are no data to support any mechanism for the spread of SARS-CoV-2 in brain. (Figs. 4, 5, 6).

## Cytokine storm

The “cytokine storm” is defined as cytokines released due to an overactive and dysregulated immune response to a different antigen, such as infections, which may lead to multiorgan dysfunction. In COVID-19, cytokine storm is associated with patients with severe illness and high concentrations of proinflammatory cytokines in plasma, especially interleukin (IL) 6, compared to moderately sick patients [204–208]. This is also associated with a poor prognosis in COVID-19 [209]. In addition, the number of white blood cells, and levels of procalcitonin and C-reactive protein are higher in the intensive care unit (ICU) cases than in non-ICU cases [204–208, 210–212]. Cytokine storm can lead to apoptosis of epithelial cells, which may lead to vascular leakage [213].

In SARS, the levels of IL-6, IL-1β, IFN, and CXCL10 are increased. These cytokines are secreted mainly by dendritic cells, neutrocytes and macrophages, indicating that the innate immunity is involved in SARS. Cytokines induce the infiltration and recruit proinflammatory cells. In SARS patients there is evidence of alveolar damage, edema and focal hemorrhage. These are also present in the manifestation of COVID-19 [214]. Since SARS-CoV and SARS-CoV-2 infect cells using mainly the same receptor (ACE2), it is possible that both viruses can affect the same immune cells and cause a cytokine storm [215–226]. Cytokines can cross the BBB, and also disrupt its integrity [149, 227–230].

## Co-morbidities increase risk of death from COVID-19: outlined

According to the CDC, 91.7% of COVID-19 confirmed hospitalized cases had at least one reported underlying medical condition. The most common preconditions associated with COVD-19 hospitalization were hypertension and obesity. However, 22% of those hospitalized had some form of neurological disease, and this was greater than hospitalized cases of asthma (~ 13%), [231]. Chronic hypertension has been linked to cerebrovascular dysfunction and deficient local perfusion [232]. Thus, it’s tempting to speculate that this significant group of patients may also include those with persistent post-infection symptoms (“long-haulers”). Further work is needed on this group of patients ([233].

There are a number of underlying conditions associated with a dysfunctional cerebrovasculature, which may make it more susceptible to SARS-CoV-2 infection and therefore, greater severity of COVID-19. While it is difficult to isolate each pre-condition as many overlaps in the patient pool, there is evidence that each one individually alters the abilities of the CNS vascular barriers to protect the brain (Fig. 5). The CNS vascular barriers are dysfunctional, both structurally and functionally with aging. The cerebrovasculature becomes leakier, with reduced capillary density, glucose transport and cerebral blood flow [105, 234]. Increased cerebrovascular MMP activities via CD147/ADAM17 breakdown the basement membrane and contribute to the vascular dysfunction [235, 236]. Proinflammatory cytokines, such as TNFα could stimulate ADAM17[69]. CD147 is expressed on the endothelium, astrocytes and astrocytes endfeet [237]. SARS-CoV-2/SP interaction with CD147 could increase MMP9 activity, which may break down the basement membrane leading to loss of pericyte coverage and tight junction proteins and thus, aggravate cerebrovascular dysfunction [101]. Hyperglycemia, as seen in diabetes, is associated with increased MMP activity [238]. Recently, it was shown that recombinant SP of SARS-CoV-2 interact with CD147 to disrupt the function of cardiac pericyte-endothelial cells [25]. Thus, MMP9 could be targeted as a possible additional therapy for COVID-19 (Fig. 6).

### Age

Age is a major risk factor for the development of a number of disorders affecting the brain, e.g., Alzheimer’s disease [105, 239–242]. As cells aged, they transition to a state of senescence, which decreases the chance of forming malignancies, and this is associated with moving to a more “inflamed state” [105, 107, 240, 243]. Aged and/or senescence cells have a myriad of changes that cause alteration in their phenotypical expression, which is associated both with an increase in circulating factors that are harmful to the CNS and decreases in factors that are protective [244]. There is an increase in cerebrovascular disruption through factors that induce caveolae-cytoskeleton interactions that result in increased fluid phase pinocytosis [245]. Aging causes a pro-inflammatory environment in the CNS, leading to reactive oxygen species (ROS), an increase in senescence cells and an increase in the number of reactive microglia and astrocytes [240]. In aging, microglia processes retract and thicken, cell bodies enlarge, and astrocytes increase expression of inflammatory surface markers [246]. This reflects a chronic, low-grade neuro-inflammation and these glia cells have a heightened response to immune stimulation. There is significant reduction in occludin expression in brain endothelium and this leads to exacerbation of neuroinflammation and leakage of harmful molecules into the brain [240, 243, 247]. Aged endothelial cells have altered expression and function of many of the important transporter and efflux proteins that help regulate the brain’s environment. Aging rodents showed reduced brain glucose uptake, and a reduction in GLUT1 expression was associated with impaired glucose utilization and neuronal damage [248]. Reduced pericytes coverage on the aging cerebrovasculature leads to its dysfunction. Efflux transporters, such as LRP-1 and P-gp, important in the clearing of toxic products from brain, are shown to be down regulated with aging [242, 249, 250].

### Obesity

According to the CDC, ~ 50% of hospitalized patients with confirmed COVID-19 were obese and ~ 28% were overweight. Over 42.0% of American adults are considered obese [231, 251]. Obesity has widespread biological changes, such as altered levels of hormones that regulate feeding (principally insulin, leptin, adiponectin and ghrelin). These hormones are known to cross the cerebrovasculature via specialized transporters [27, 135]. Obesity is linked to increase in neuroinflammation and neuronal loss in areas of the brain (arcuate nucleus and lateral hypothalamus) that have a high metabolism [109, 114, 252]. In mice fed a high fat diet (HFD), there is disruption of the cerebrovasculature and tanycytes expression of transporters [252]. HFD increased activation of astrocytes and microglia, decreased tight junction proteins (claudins-5 and -12), increased permeability and downregulated cytoskeleton proteins (vimentin and tubulin) [109, 253–255]. HFD increased leukocytes penetration across the vasculature as well as increased inflammatory cytokine production, which can cross the vasculature leading to a cycle of neuroinflammation, decrease in barrier properties and increasing its damage.

### Diabetes Mellitus

Metabolic disorders as an underlying condition increased the prevalence of COVID-19 confirmed hospitalizations [231, 256]. In the USA, ~ 11% of Americans have diabetes mellitus (DM). Neurological and cognitive issues, such as dementia, as well as vascular disorders, such as stroke, are documented in DM. Diabetic encephalopathy, a progressive complication of diabetes, has been linked to the increase probability of neurological related disorders. These are related to macrovascular and microvascular injury due to endothelial dysfunction [257, 258]. In vitro and in vivo studies have shown that hyperglycemia is a primary driver for detrimental effects on vascular endothelial cells and microvascular injury underlies the classic pathology of DM [259]. Vascular damage seems to occur from excess glucose concentration within the endothelial cells, causing excessive ROS and damage to mitochondria [257, 258]. The NVU shows sign of modification due to hyperglycemia, with increase in basement membrane thickness, pericyte degeneration and increase in markers in the brain parenchyma due to barrier breakdown [258]. Collectively, these changes at the CNS vascular barriers could aggravate SARS-CoV-2 cerebral effects.

### Hypertension

Hypertension is associated with an increased incidence of confirmed COVID-19 cases [231]. The cerebrovasculature has been shown to be disrupted in cardiovascular disease, such as neurogenic hypertension [10, 260]. The RAS system is a major contributor to cardiovascular disease and hypertension. Angiotensin II has been shown to induce oxidative stress and inflammation, and appears to precede hypertension [10]. In hypertensive rats, there are decreased levels of the tight junction components (occludin and ZO-1). This hypertension is related to chronic, low grade inflammation in the brain and the release of circulating inflammatory molecules [10, 260]. Circulating inflammatory factors and inflammatory cells could enter the CNS and contribute to the adverse effects. In addition, astrocytes and microglia can release a myriad of chemokines and cytokines, which can increase the recruitment of leukocytes and increase vascular disruption [260] (Fig. 6).

## Conclusions

There are many possible ways the virus can affect the brain. The main way is due to dyspnea and thromboembolism that deprive the brain of oxygen and blood, and can lead to cerebral edema. Depending on the viral load, it can enter brain via retrograde transport along nerve endings in the buccal and nasal cavities, the main route of entry of the virus into the body. While the levels of SARS-CoV-2 in blood is low, from blood the virus can enter the brain via the normal or dysfunctional vasculature and access cells within brain, which express receptors that can interact with this virus. Interaction with ACE2 and/or CD147 on cells of the NVU could activate MMP9 that would break down the basement membrane, and render pericytes dysfunctional. The SP can slowly cross the cerebrovasculature via absorptive transcytosis, but not by ACE2. Entry into the CSF via the choroid plexus could distribute the virus to brain along the CSF drainage route. Virus could also enter brain with the trafficking of leukocytes. Thus, the aging brain may be a neurovascular target of SARS-CoV-2 with potentially devastating consequences, but there is no sound evidence of major neuroinvasion. In addition, the significance of neuroinvasion to the disease outcome in unclear since there is overwhelming respiratory failure in patients from which brain samples were tested for SARS-CoV-2. A long-term effect of this viral infection may be neurodegeneration and behavioral changes. A better understanding of the SARS-CoV-2 on the aging vasculature could help in health care management of these susceptible patients and assist in the development of therapies that target the virus to prevent severe outcomes from COVID-19.

## Data Availability

Not applicable as this is a review manuscript.

## Abbreviations

ACE: Angiotensin converting enzyme
ACE2: Angiotensin converting enzyme (ACE) 2 receptors
ARDS: Acute respiratory distress syndrome
ADAM17: A disintegrin and metalloprotease17
ANE: Acute necrotizing encephalopathy
AT1: Angiotensin receptor 1
BBB: Blood–brain barrier
BP: Blood pressure
CD147: EMMPRIN (extracellular matrix metalloprotease inducer)
CDC: Enter of Disease Control
Cov: Coronavirus
COVID-19: Coronovirus December 2019
CSF: Cerebrospinal fluid
CNS: Central nervous system
CT: Computer tomography
HCoV-NL63: Human coronavirus NL63
HCoV-2295: Human coronavirus 2295
HFD: High fat diet
GLUT1: Glucose transporter 1
LRP-1: Low- density lipoprotein-related receptor 1
MERS-CoV: Middle East Respiratory Syndrome CoV;MMP9:matrix metalloprotease 9;NVU: neurovascular unit
PECAM-1: Platelets endothelial cell adhesion molecule-1 (CD31)
RAGE: Receptor for advanced glycation end products
RAS: Renin angiotensin system
RNA: Ribonucleic acid
RT-PCR: Reverse transcription polymerase chain reaction
SARS: Severe acute respiratory syndrome
SARS-CoV: Severe acute respiratory syndrome-CoV
SARS-CoV-2: Severe acute respiratory syndrome-CoV-2
ROS: Reactive oxygen species
TMPRESS 2: Transmembrane protease, serine
